# Synergistic protective effects of TCM formula NRICM102 and *N*-acetylcysteine against hepatorenal injury in a mouse model of bongkrekic acid poisoning

**DOI:** 10.3389/fphar.2025.1596785

**Published:** 2025-06-05

**Authors:** Yuh-Chiang Shen, Yea-Hwey Wang, Kuo-Tong Liou, Wen-Chi Wei, Jing-Jy Cheng, Hui-Kang Liu, Nai-Kuei Huang, I-Wen Lo, Cher-Chia Chang, Wen-Fei Chiou, Keng-Chang Tsai, Chun-Tang Chiou, Chia-Ching Liaw, Yi-Chang Su

**Affiliations:** ^1^ National Research Institute of Chinese Medicine, Ministry of Health and Welfare, Taipei, Taiwan; ^2^ School of Nursing, National Taipei University of Nursing and Health Science, Taipei City, Taiwan; ^3^ Department of Medicine, Mackay Medical College, Taipei, Taiwan; ^4^ Department of Chinese Medicine, Tri-Service General Hospital, National Defense Medical Center, Taipei, Taiwan; ^5^ Institute of Pharmacology, School of Medicine, National Yang Ming Chiao Tung University, Taipei, Taiwan; ^6^ Program in Medical Biotechnology, College of Medical Science and Technology, Taipei Medical University, Taipei, Taiwan; ^7^ Department of Biochemical Science and Technology, National Chiayi University, Chiayi, Taiwan; ^8^ Department of Pharmacy, School of Pharmaceutical Sciences, National Yang Ming Chiao Tung University, Taipei, Taiwan; ^9^ Graduate Institute of Natural Products, Kaohsiung Medical University, Kaohsiung, Taiwan

**Keywords:** Bongkrekic acid, multi-organ failure, NRICM102, *N-acetylcysteine*, food poisoning

## Abstract

Bongkrekic acid (BKA), a mitochondrial toxin produced by *Burkholderia cocovenenans* subsp. *farinofermentans*, is typically found in contaminated fermented rice products such as tempeh bongkrek, causing severe foodborne illnesses marked by systemic inflammation, multi-organ failure (MOF), and high mortality rates (40%–100%). A recent outbreak in Taiwan (2024) resulted in six fatalities among 33 affected individuals, underscoring the urgent clinical need for effective treatments. This study evaluated the therapeutic potential of NRICM102, a novel traditional Chinese medicine (TCM) formulation, combined with the antioxidant *N*-acetylcysteine (NAC), against BKA-induced hepatorenal toxicity in a mouse model. NRICM102 (1.5–3.0 g/kg), NAC (0.5 g/kg), and their combination significantly improved survival, reduced serum biomarkers (GOT, GPT, BUN), and alleviated liver and kidney histopathological damage following acute (5.0 mg/kg) and subacute (2.0 mg/kg) BKA exposure. RNA-seq analyses suggested that the NRICM102-NAC combination synergistically modulated critical pathways, including mitochondrial function, cytochrome P450 enzyme activity, oxidative stress, immune responses, and cell death regulation. Despite these promising findings, mechanistic conclusions remain associative and require further validation using targeted mitochondrial studies. Collectively, NRICM102 combined with NAC offers a promising, translationally relevant therapeutic strategy warranting additional preclinical safety and pharmacokinetic assessments to advance toward clinical application.

## 1 Introduction

Bongkrekic acid (BKA) is a rare mitochondrial toxin produced by Burkholderia cocovenenans subsp. farinofermentans, commonly found in contaminated fermented rice flour or tempeh bongkrek, a traditional Indonesian food made from coconut milk and beans (Anwar et al., 2017). BKA has been responsible for multiple severe foodborne illness outbreaks globally, including a recent incident in Taiwan in 2024, affecting 33 individuals and resulting in six fatalities (Su, 2024; Vandamme et al., 1996). Mortality rates from BKA poisoning range from 40% to 100%, driven by rapid progression to systemic inflammation and multi-organ failure (MOF), particularly impacting liver and kidney function. Despite its severe clinical outcomes, there are currently no specific therapeutic interventions available, leaving medical care limited to supportive treatments and underscoring a critical need for effective therapeutic strategies.

Although outbreaks are sporadic, BKA poisoning has occurred globally, including in Africa, China, Southeast Asia, and Taiwan, consistently exhibiting high fatality rates (Han et al., 2023). The pathogenic mechanisms of BKA poisoning are primarily attributed to its ability to inhibit mitochondrial adenine nucleotide translocase (ANT), a protein crucial for ATP/ADP exchange, leading to mitochondrial dysfunction, apoptosis, and cell death (Anwar et al., 2017; Li et al., 2023). However, considerable mechanistic knowledge gaps remain regarding how BKA-mediated mitochondrial impairment specifically triggers systemic inflammatory responses and multi-organ toxicity. Addressing these gaps is essential for developing targeted therapies.

NRICM102, a novel traditional Chinese medicine (TCM) formulation, was initially developed and utilized successfully during the COVID-19 pandemic in Taiwan to alleviate pulmonary embolism and fibrosis associated with MOF, significantly reducing mortality (Tseng et al., 2022; Wei et al., 2022). Previous research has demonstrated its capacity to modulate critical inflammatory pathways, including TLR, JAK/STAT, PI3K/AKT signaling, and neutrophil extracellular trap (NET) formation, as well as mitigate cytokine storms. Despite its traditional roots, the mechanistic understanding and application of NRICM102 in toxin-induced mitochondrial dysfunction and inflammation remain unexplored, presenting a novel therapeutic potential beyond its established use.

Considering the oxidative stress and apoptosis induced by BKA-driven mitochondrial damage, we further evaluated the therapeutic efficacy of NRICM102 combined with *N*-acetylcysteine (NAC), a well-established antioxidant known to reduce reactive oxygen species (ROS), inhibit apoptosis, and improve cell viability (Zhang et al., 2011; Zhitkovich, 2019). NAC has demonstrated synergistic benefits in various oxidative stress-mediated conditions, including enhanced protection against acetaminophen-induced hepatic injury when combined with other antioxidants (Yalcin et al., 2008; Raghu et al., 2021). Therefore, this study investigates the combined therapeutic potential of NRICM102 and NAC as an innovative approach to addressing the mechanistic and clinical challenges of BKA-induced toxicity.

## 2 Materials and methods

### 2.1 Chemicals and reagents

Bongkrekic acid (BKA) was obtained from Sigma-Aldrich (MO, United States). Tetramethylrhodamine ethyl ester (TMRE) was obtained from Invitrogen (Thermo Fisher Scientific (Grand Island, NY, United States). Chambered cover glasses were purchased from ibidi (Fitchburg, WI, United States). DMEM and fetal bovine serum (FBS) were obtained from Gibco/Thermo Fisher Scientific (Grand Island, NY, United States). All chemicals, except those indicated otherwise, were purchased from Sigma-Aldrich (MO, United States).

### 2.2 Plant materials

Houttuyniae Herba (Saururaceae; *Houttuynia cordata* Thunb.), Poria (Polyporaceae; *Wolfiporia extensa* (Peck) Ginns), Trichosanthis Fructus (Cucurbitaceae; *Trichosanthes kirilowii* Maxim), Artemisiae Herba (Compositae; *Artemisia capillaris* Thunb.), Scutellariae Radix (Labiatae; *Scutellaria baicalensis* Georgi), Polygonati Odorati Rhizoma (Liliaceae; *Polygonatum odoratum* (Mill.) Druce), Glycyrrhizae Radix et Rhizoma (Leguminosae; *Glycyrrhiza glabra* L.), Magnoliae Cortex (Magnoliaceae; *Magnolia officinalis* Rehder & E.H.Wilson), and Pinelliae Rhizoma (Araceae; *Pinellia ternata* (Thunb.) Makino), Aconiti Lateralis Radix Praeparata (Ranunculaceae; *Aconitum carmichaelii* Debeaux) were provided from by the TCM pharmacies of Taichung Veterans General Hospital in Taiwan. All TCM materials identified by Dr. Chia-Ching Liaw, the curator of NRICM herbarium and these voucher specimens (NRICM-LC2020012∼21) were deposited at NRICM, Taipei, Taiwan.

### 2.3 Preparation and HPLC analysis of NRICM102

NRICM102 was prepared and subjected to HPLC analysis, following the method outlined by the previous literature (Wei et al., 2022). It consisted of 10 TCM botanical drugs, including Houttuyniae Herba (37.50 g), Poria (18.75 g), Trichosanthis Fructus (18.75 g), Artemisiae Herba (18.75 g), Scutellariae Radix (11.25 g), Polygonati Odorati Rhizoma (11.25 g), Glycyrrhizae Radix et Rhizoma (7.50 g), Magnoliae Cortex (11.25 g), Pinelliae Rhizoma (11.25 g), and Aconiti Lateralis Radix Praeparata (7.50 g). The formula was boiled in 1,200 mL of water and simmered to reduce to 300 mL, then the decoction further was dried by freeze-dried to obtain the sample (17.7 ± 0.5 g per 300 mL). HPLC was performed on a Shimadzu Nexera-*i* LC-2050C 3D Liquid Chromatograph (Shimadzu, Kyoto, Japan) equipped with an COSMOSIL 5C_18_-AR-II column (ID 4.6 mm × 250 mm).

The HPLC profile employed a mobile phase of D.D. H_2_O with 0.3% phosphoric acid (PA) and acetonitrile (ACN) using a gradient condition following as 0.01–5.00 min, 0%–0% ACN; 5–10 min, 0%–3% ACN; 10–15 min, 3%–10% ACN; 15–25 min, 10%–15% ACN; 25–30 min, 15%–20% ACN, 30–35 min, 20%–20% ACN, 35–50 min, 20%–40% ACN, 50–60 min, 40%–100% ACN. The other HPLC parameters were set at a flow rate of 1.0 mL/min, sample concentration of 10 mg in D.D. H_2_O, an injection volume of 10 μL, and the column oven at 40°C. Twenty plant metabolites were isolated using an open column and preparative HPLC NRICM102, and their chemical structures were identified using NMR and high-resolution electrospray ionization mass spectrometry. An HPLC fingerprint of NRICM102 was established and shown in Supplementary Figure S1.

### 2.4 BKA-induced acute and subacute mice model

For the acute and subacute BKA poisoning mouse models, male ICR mice, aged 6–8 weeks and weighing between 22 and 25 g, were sourced from the National Laboratory Animal Breeding and Research Center in Taipei, Taiwan. The mice were acclimatized for a period of 1 week under controlled environmental conditions, specifically maintained at 22–24°C with a humidity level of 55%–60%. The light-dark cycle was set to 12 h, and the mice had *ad libitum* access to standard chow (MFG, Oriental Yeast Co., Japan) and water. Each model, both acute and sub-acute, consisted of six treatment groups (n = 10 per group), assigned in a randomized, double-blind manner: 1) Control (saline), 2) BKA + saline, 3) BKA + NRICM102 (1.5 g/kg), 4) BKA + NRICM102 (3.0 g/kg), 5) BKA + NAC (0.5 g/kg), and 6) BKA + NRICM102 (3.0 g/kg) + NAC (0.5 g/kg). A single oral dose of BKA (2.0 (in subacute phase) or 5.0 mg/kg (in acute phase)) was administered, followed 15 min later by NRICM102 (1.5 or 3.0 g/kg or NAC (0.5 g/kg). Serum and tissue samples were collected at designated time points [at 5 h after BKA (5.0 mg/kg) administration or on day 1, 3, 14, and 21 after BKA (2.0 mg/kg) administration]. For the subacute model, mice were fasted for 12 h before blood collection under isoflurane anesthesia on the final day. Liver and kidney tissues were harvested post-euthanasia (CO_2_), and BUN and SCr levels were measured using Fuji Dri-Chem Slide kits (Fujifilm, Japan).

### 2.5 Immunohistochemistry and histopathology examination

For immunohistochemical staining, 15–20 consecutive liver sections (20–30 μm thick) were collected from each group. All the sections were fixed, permeabilized, blocked, and randomly selected for specific marker staining. Primary antibodies were diluted in PBS with 3% albumin and incubated overnight at 4°C. Antibodies targeting nitrotyrosine (NT; 1:100), myeloperoxidase (MPO) (1:100), IL-1β (1:100), NLRP3 (1:100), CD14 (1:100), CCL6 (1:50), and CCR1 (1:50) were obtained from GeneTex (Irvine, CA, United States). Antibodies against MAC-1 (CD11b, 1:50), CitH3 (NET, 1:50), and CD106 (VECAM-1, 1:50) were obtained from Abcam (Cambridge, United Kingdom); Cleaved Casp3 (Cap3; 1:50) was obtained from BD Pharmingen (San Diego, CA, United States). After washing, the sections were incubated with Alexa Fluor^®^-conjugated secondary antibodies (488, 555, or 647; Cell Signaling Technology) and counterstained with DAPI to visualize the nuclei. Stained sections were analyzed using a Zeiss LSM780 confocal microscope. Immunopositive areas and cell distributions were quantified as relative stained areas (%) using Zen 2011 software (Carl Zeiss) and AlphaEase FC (Alpha Innotech) across regions of interest at 100–1×50 magnification, with data collected from three to five independent experiments. Histological analysis and fibrosis detection were performed using H&E and Masson’s trichrome staining, with section images captured using a microscope and analyzed for staining intensity using ImageJ software.

### 2.6 RNA sequencing (seq) and RNA-seq data analysis

TRIzol, a reliable RNA extraction reagent, was used to isolate the total RNA from all biological samples immediately after the experiment. A SimpliNano™ spectrophotometer (Biochrom, MA, United States) was used to determine the purity and quantity of each RNA sample. RNA degradation and integrity were monitored using a Qsep 100 DNA/RNA Analyzer (BiOptic Inc., Taiwan). Sequencing libraries were generated from the total RNA of the samples using a KAPA mRNA HyperPrep Kit (KAPA Biosystems, Roche, Basel, Switzerland). High-throughput sequencing (Illumina NovaSeq 6000 platform) was performed to obtain raw data, and Fast QC and MultiQC were used to assess quality. High-quality raw paired-end read data were obtained using Trimmomatic (v = 0.38) for subsequent analysis. The alignment of read pairs from each sample to a reference genome was performed using the HISAT2 software (v 2.1.0), after which the reads mapped to individual genes were counted in FeatureCounts (v 2.0.0). Differentially expressed gene (DEG) analysis of the case and control samples was performed using DEGseq (v. 1.40.0) or DESeq2 (v. 1.26.0). Gene Ontology (GO) functional annotation and Kyoto Encyclopedia of Genes and Genomes (KEGG) pathway enrichment analyses were performed using clusterProfiler (v. 3.14.3). All data of RNA sequencing have been uploaded.

### 2.7 Mitochondrial membrane potential measurement

H4IIE cells (rat liver-derived) was selected to assess hepatic toxicity and H9C2 cells (rat cardiac-derived) to explore potential cardiac mitochondrial damage, allowing comprehensive evaluation of NRICM102/NAC’s organ-protective effects. H9C2 cells (3.5 × 10^4^) or H4IIE cells (1 × 10^4^) were seeded on chambered cover glasses. The cells were then pre-treated with NRICM102 (50 μg/mL) or NAC (4 mM) for 2 h. After pretreatment, BKA (50 μg/mL) was added and the cells were incubated at 37°C for 48 h. In a separate experiment with rat hepatoma, H4IIE cells (ATCC; 3 × 10^3^ cells/well) were seeded into 96-well plates and allowed to adhere overnight. NRICM102 was extracted by boiling and freeze-drying, then reconstituted in sterile water and filtered before use. Treatments included vehicle control (0.1% DMSO), BKA (10 μg/mL) alone, BKA with NRICM102 (35–140 μg/mL) or NAC (2 mM) for 72 h. Following treatment, cells were washed with Hank’s Buffered Salt Solution and stained with 100 nM TMRE for 15 min to assess mitochondrial membrane potential via fluorescence intensity. Imaging was performed using an LSM900 microscope (Carl Zeiss, Göttingen, Germany) under fixed gain settings. The fluorescence intensity per cell was quantified using the ImageJ software after background subtraction. For each treatment condition, at least four frames were captured, with 10–15 cells per field analyzed for quantification (Deng et al., 2024).

### 2.8 Statistical analysis

GraphPad Prism (version 9.0; GraphPad Software, San Diego, CA) was used for data analysis. The results are presented as the mean ± S.E.M. Statistical analysis involved one-way ANOVA followed by the S-N-K t-test. Differences were considered statistically significant at *p* <0.05.

## 3 Results

### 3.1 NRICM102 in combination with NAC reduces high-dose BKA-induced acute hepatotoxicity and increases survival in mice

**FIGURE 1:**
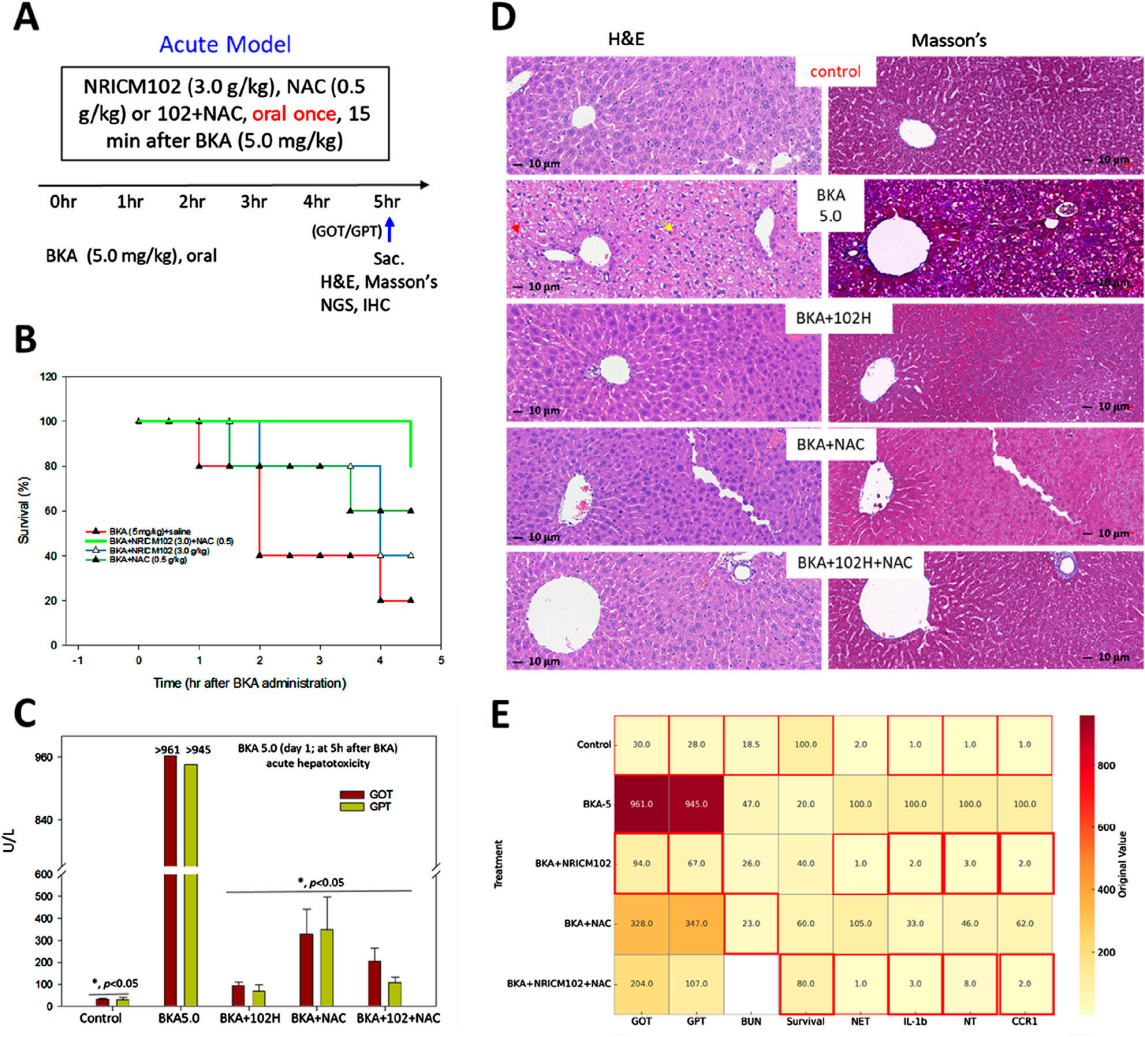
Effects of NRICM102 and NAC on hepatotoxicity induced by high-dose BKA (5.0 mg/kg) over 4.5 h. **(A)** Experimental design of the acute BKA poisoning mouse model. Mice were administered a single oral dose of BKA at a dosage of 5.0 mg/kg. This was followed 15 min later by the administration of NRICM102 at doses of 3.0 g/kg, either as a standalone treatment or in combination with NAC at a dosage of 0.5 g/kg. **(B, C)** Survival rates and liver enzyme levels (GOT and GPT) were assessed at indicated time points, with GOT and GPT measured at 4.5 h. Reductions were statistically significant (*p < 0.05) when compared to the control or the BKA-only group on day 1 (n = 10, each group). **(D)** Typical H&E staining of liver tissue in the BKA + saline group (BKA5.0) shows extensive hepatocyte necrosis with vacuolation, pyknotic nuclei (red arrows), and hepatic sinus congestion (yellow arrows). Masson’s staining reveals significant tissue fibrosis (blue) in the BKA (5.0) group, which is notably reduced in the other treatment groups. **(E)** *Heatmap of original biomarker values across treatment groups with optimal value highlights.* This heatmap visualizes the original (unnormalized) values of biochemical and inflammatory markers (GOT, GPT, BUN, NET, IL-1b, NT, CCR1) and survival rate in different treatment groups following bongkrekic acid (BKA) exposure. Each cell displays the raw value corresponding to a specific treatment and biomarker. Red borders indicate optimal values for each parameter: for Survival, the highest value is highlighted; for all other markers (GOT, GPT, BUN, NET, IL-1b, NT, CCR1), the lowest values are considered optimal and thus highlighted. ND: not determined. Red borders highlight the optimal values.

To evaluate the combined therapeutic effects of NRICM102 and NAC in acute BKA poisoning, mice received a high-dose BKA challenge (5.0 mg/kg) followed 15 min later by a single oral administration of NRICM102 (1.5 or 3.0 g/kg), NAC (0.5 g/kg), or their combination [NRICM102 (3.0 g/kg) plus NAC (0.5 g/kg)] (Figure 1A). High-dose BKA alone resulted in severe hepatotoxicity and rapid mortality, with 80% of untreated mice died within 4.5 h (Figure 1B) and liver enzyme levels (GOT and GPT) exceeding 900 U/L (Figure 1C). Individual treatments with NRICM102 or NAC significantly lowered liver enzyme concentrations and improved survival to approximately 80% within 3–4 h. The NRICM102-NAC combination further enhanced survival rates (80%–100%) at 4–4.5 h and markedly reduced hepatotoxicity. In addition, all treatment groups showed significantly reduced liver-to-body weight ratios compared to the BKA-only group (*P* <0.05, Supplementary Table S1). Histological examination revealed extensive hepatocyte necrosis, vacuolation, pyknotic nuclei (red arrows), hepatic sinus congestion (yellow arrows), and collagen deposition in BKA-treated livers (Figure 1D), consistent with previous reports (Shi et al., 2019). These pathological changes were significantly alleviated by NRICM102 or NAC alone, and further improvement was observed with combined NRICM102+NAC treatment. A heatmap visualizes the original (unnormalized) values of biochemical and inflammatory markers (GOT, GPT, BUN, NET, IL-1β, NT, CCR1) and survival rate in different treatment groups following BKA exposure (Figure 1E).

### 3.2 The effects of NRICM102 or NAC on attenuation of subacute BKA-induced hepatorenal toxicity in mice

**FIGURE 2:**
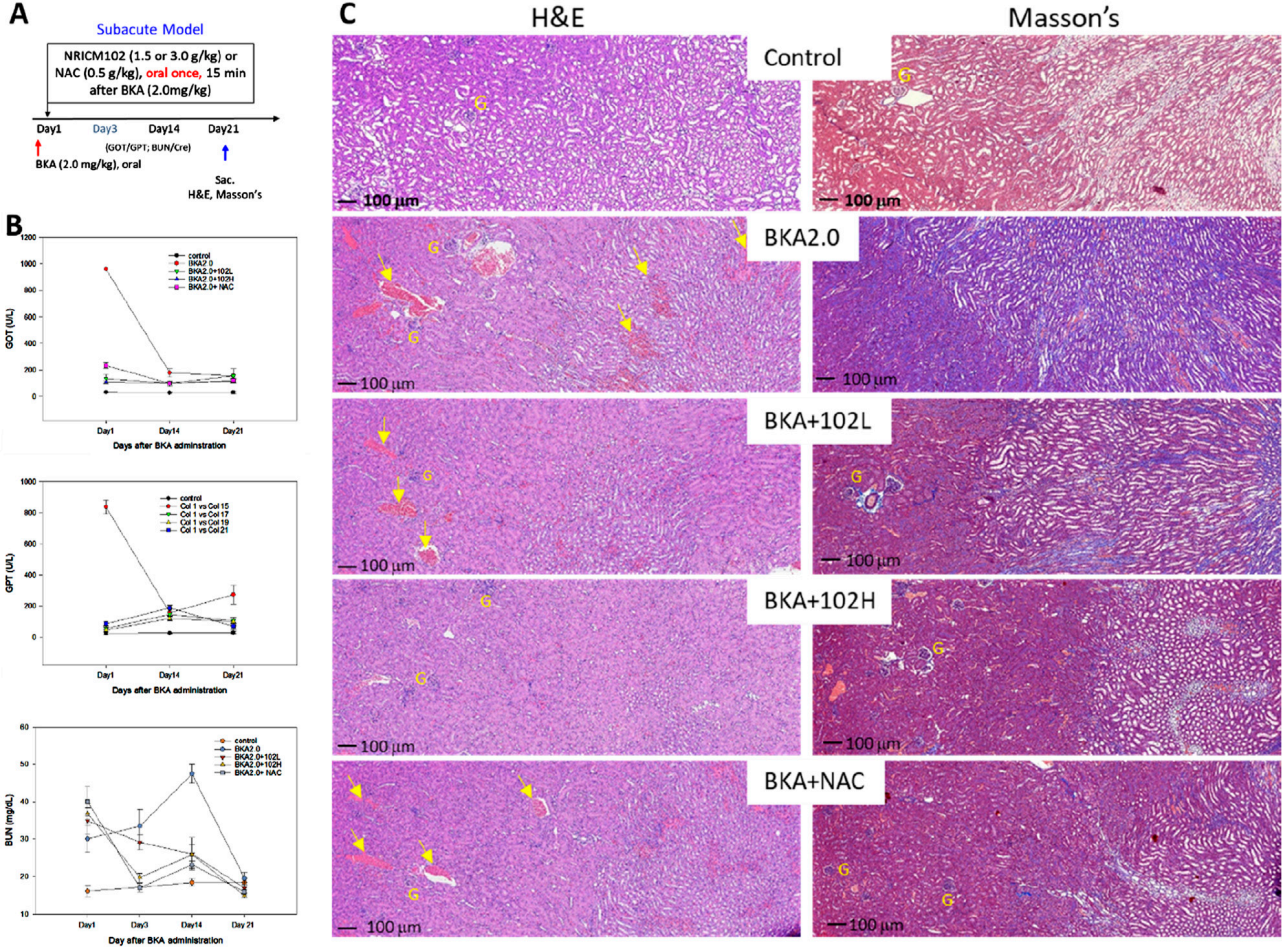
Effects of NRICM102 and NAC on hepatotoxicity and renal toxicity induced by subacute dosing of BKA. **(A)** Experimental design of acute BKA poisoning mouse model. Mice received a single oral dose of BKA (2.0 mg/kg), followed 15 min later by NRICM102 (1.5 or 3.0 g/kg), either alone or in combination with NAC (0.5 g/kg). **(B)** Serum samples were analyzed for (U/L), GPT (U/L), and blood urea nitrogen (BUN) levels (mg/dL) on days 1, 3, 14, and 21. The reductions observed were statistically significant (†,*p < 0.05) when compared to the control group (†) and the BKA group (*) on day 1, respectively. The analysis included a total of 10 subjects in each group. **(C)** In the BKA-treated group, typical H&E staining showed necrosis of the proximal tubules and protein casts within renal tubules were visible (yellow arrows, left panel). Typical Masson’s staining showed increased collagen deposition (blue) in the BKA group, indicating fibrosis (right panel). Scale bar: 100 μm; G denotes glomerulus. Group: Treatment with low-dose NRICM102 (1.5 g/kg; BKA2.0 + 102L), high-dose NRICM102 (3.0 g/kg; BKA2.0 + 102H), or NAC (0.5 g/kg; BKA2.0+NAC) effectively reduced these enzyme levels compared to the BKA-only group.

We established a subacute mouse model of BKA poisoning by orally administering a single dose of BKA (2 mg/kg) to evaluate the combined therapeutic effects of NRICM102 and NAC. Fifteen min after BKA administration, mice received a single oral dose of NRICM102 (1.5 or 3.0 g/kg) or NAC (0.5 g/kg) (Figure 2A). No mortality occurred in any group during the 21-day observation period. Additionally, no significant differences in body weight were noted among groups (Supplementary Table S2). In mice treated with BKA plus saline, acute liver injury was evident, with serum GOT and GPT levels peaking on day 1 and decreasing gradually to around 200 U/L by days 14–21. Treatment with NRICM102 or NAC significantly attenuated the initial elevation in GOT and GPT levels, effectively reducing hepatic damage by day 21 (Figure 2B). Similarly, subacute kidney injury induced by BKA, indicated by elevated blood urea nitrogen (BUN) levels peaking on day 14 and returning to baseline by day 21, was significantly alleviated by NRICM102 (1.5 or 3.0 g/kg) or NAC treatment (Figure 2B, *P* < 0.05). Kidney histology on day 21 showed proximal tubule necrosis and protein casts in BKA-treated mice, along with increased collagen deposition revealed by Masson’s staining (Figure 2C). These pathological alterations were notably mitigated by treatment with NRICM102 (1.5 or 3.0 g/kg) or NAC (0.5 g/kg).

### 3.3 NGS analysis uncovers molecular mechanisms and pathways in NRICM102s protective effects against BKA-induced hepatotoxicity

**FIGURE 3:**
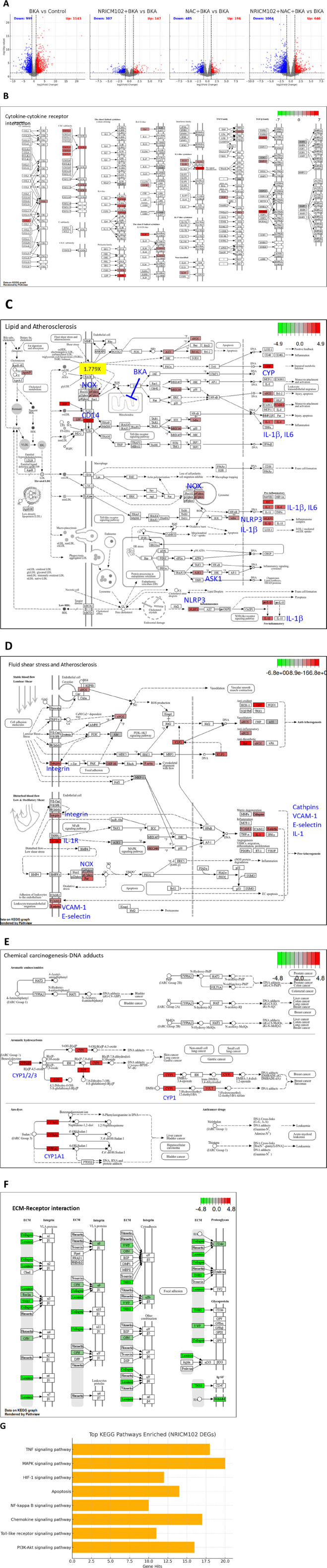
NGS Analysis of NRICM102 effects on BKA (5.0)-induced liver tissue. **(A)** Volcano plots depict differentially expressed genes (DEGs) across treatment groups. **(B–E)** KEGG analysis revealed upregulation of **(B)** cytokine–cytokine interaction, **(C)** lipid metabolism and atherosclerosis, **(D)** fluid shear stress and atherosclerosis, and **(E)** DNA damage response in the BKA-treated group compared to the control group. **(F)** The extracellular matrix (ECM)-receptor interaction pathway was downregulated in the BKA + NRICM102+NAC group compared to the BKA-treated group. **(G)** The bar chart illustrates the top signaling pathways significantly enriched among DEGs identified from the NRICM102 treatment group. Prominent pathways include the TNF signaling pathway, MAPK signaling pathway, PI3K-Akt signaling pathway, and Toll-like receptor signaling pathway, all of which are critical in inflammation, apoptosis, immune regulation, and cell survival. The x-axis indicates the number of DEGs involved in each pathway.

NRICM102 and/or NAC treatment exhibited the significant potential for attenuation of BKA-induced hepatotoxicity as indicated by GOT, GPT, and histological analysis. To further investigate the potential underlying mechanism, we examined transcriptional changes in liver tissue using RNA-Seq analysis of mRNA expression. Differentially Expressed Genes (DEGs) between the groups were identified using a significance threshold of *p* < 0.05 and |log_2_FoldChange| > 1. As shown in Figure 3A, a total of 2,142 DEGs were identified between the control group and the group treated with BKA. Among these, 1,143 genes were upregulated, while 999 genes were downregulated. The treatment with NRICM102 alone resulted in 454 DEGs, consisting of 147 upregulated and 307 downregulated genes compared to the BKA group. Additionally, treatment with NAC alone resulted in the identification of 681 DEGs, consisting of 196 upregulated and 485 downregulated genes. Notably, the combination treatment of NRICM102 and NAC showed the most significant results, with a total of 1,410 DEGs identified, which included 646 upregulated and 764 downregulated genes.

Further analysis using the Kyoto Encyclopedia of Genes and Genomes (KEGG) revealed pathways related to cytokine and receptor interactions (Figure 3B), lipid metabolism and atherosclerosis (Figure 3C), fluid shear stress and atherosclerosis (Figure 3D), as well as DNA damage due to chemical exposure (Figure 3E) were elevated in BKA-treated group compared to control-group. Notably, the combination treatment of NRICM102 and NAC showed significant effects in several pathways impacted by BKA treatment, including a marked reduction in the expression of the extracellular matrix (ECM)-receptor interaction pathway (Figure 3F). Additionally, DEGs analysis indicated that treatment with NRICM102, NAC, or their combination significantly modulated key genes associated with inflammation (IL-1β, IL-6, p67phox, and CD14), cell death (NLRP3 and IL-1β), immune response activation (NET and MPO), chemokine signaling and receptor activity (CCL6, CXCL14, CCR1, and CXCR2), liver metabolism (CYP1/2/3), fibrosis (MMP-9), and atherosclerosis (VCAM-1, E-selectin, Integrin) (Table 1).

**TABLE 1:** Analysis of differentially expressed genes (DEGs) related to inflammation in BKA-administered mice treated with NRICM102, NAC, or their combination.

Gene symbol	Log_2_ fold change			
	BKA/control	BKA + 102/BKA	BKA + NAC/BKA	BKA + 102 + NAC/BKA
*IL-1*β	2.66	−1.73	−1.77	−3.58
*IL-6*	2.53	−1.38	NA^a^	−2.73
*p67(phox)*	1.64	−1.11	−0.81	−1.51
*CD14*	4.01	−1.95	−1.33	−3.48
*NLRP3*	2.26	−1.82	−1.42	−2.11
*Mac-1 (CD11b)*	1.37	−1.69	−1.80	−1.10
*vWF*	1.59	−0.98	−0.89	−2.12
*CCL6*	3.59	−1.41	−0.96	−1.78
*CXCL14*	2.35	−2.35	−2.51	−4.00
*CCR1*	2.75	−2.43	−0.99	−2.02
*CXCR2*	2.85	−2.70	−1.38	−2.20
*VCAM-1*	2.32	−1.19	−0.73	−1.77
*MMP-9*	2.24	−2.41	−2.44	−2.43

^a^ NA: data not available. Italic symbols with underline indicate that the combined effect of NRICM102 and NAC, is more effective in counteracting the effects of BKA, than either NRICM102 or NAC, alone.

Enrichment analysis of NRICM102-regulated DEGs (148 genes in DEGs table with |log2FoldChange| > 1.5, *p*-value <0.05) revealed significant modulation of immune, inflammatory, and cellular stress response pathways. Notably, the TNF signaling pathway and MAPK signaling pathway were highly enriched in KEGG analysis (Figure 3G), suggesting a key role of NRICM102 in modulating inflammation and cell survival signaling. In parallel, GO Biological Process enrichment indicated prominent involvement in regulation of inflammatory response, response to oxidative stress, and positive regulation of apoptosis. Overall, these findings suggest that NRICM102 exerts protective effects against BKA-induced toxicity primarily through anti-inflammatory mechanisms, stress response modulation, and apoptosis regulation.

**FIGURE 4:**
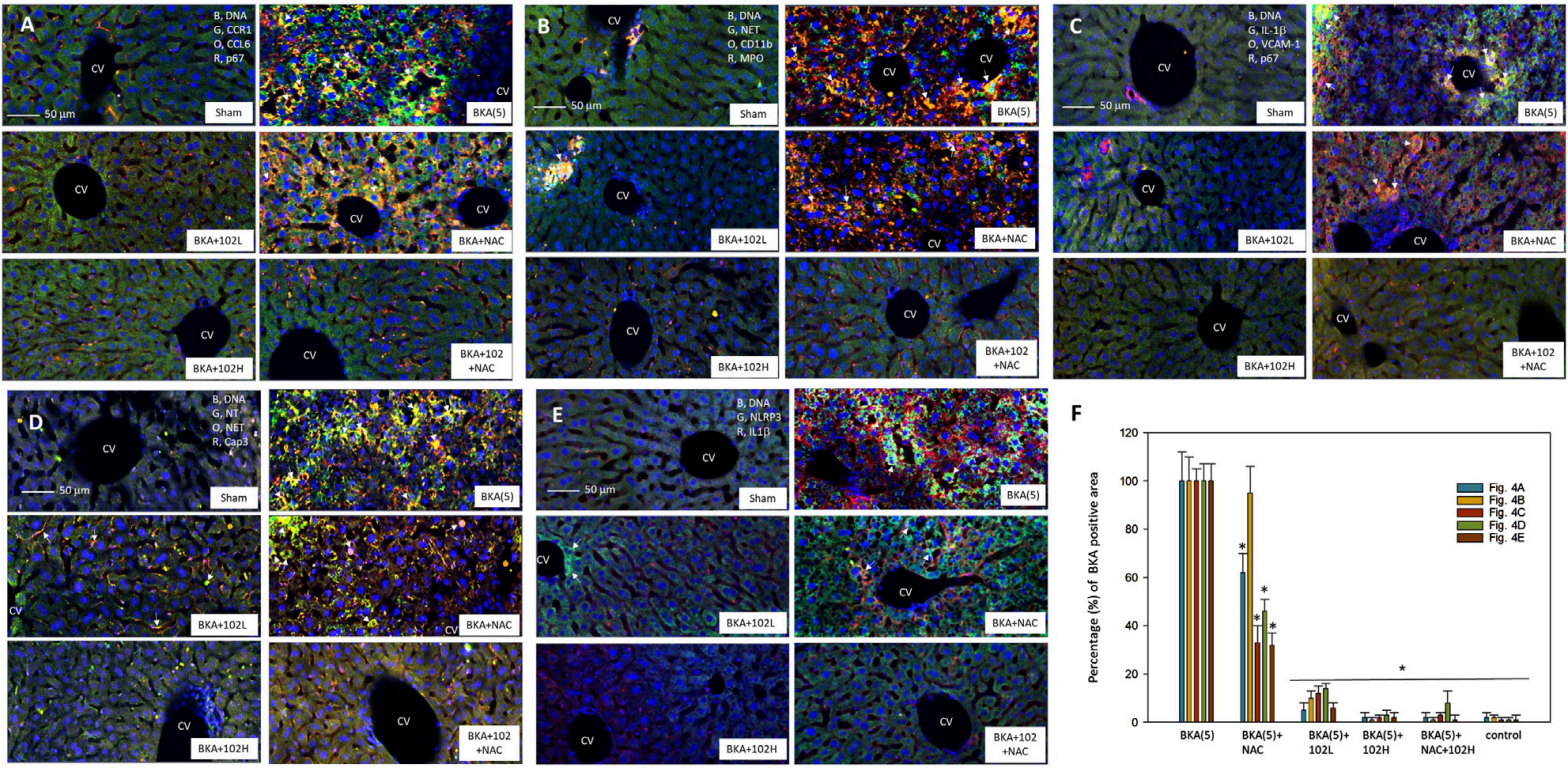
Effects of NRICM102 and NAC on inflammation markers in acute BKA-induced liver injury (4.5 h post-treatment). Confocal images show colocalization of inflammation markers in liver tissue: **(A)** CCR1 (green) and p67phox (red) colocalized with CCL6 (orange), indicated by arrows. **(B)** CD11b (orange) aligns with NET formation (green) and myeloperoxidase (MPO, red); arrows highlight colocalization of CD11b and MPO. **(C)** IL-1β (green) colocalized with VCAM-1 (orange) and p67 (red), as indicated by arrows. **(D)** Cleaved caspase-3 (Cap3, red) overlapped with nitrotyrosine (NT, green) and NET (orange); arrows indicate colocalization of cCasp3 with NET or NT. **(E)** NLRP3 (green) colocalized with IL-1β (red), highlighted by arrows. **(F)** Quantification of BKA-positive staining areas (%), presented as mean ± S.E.M. (n = 3 per group). Experimental groups are as in Figure 4. *p < 0.05 vs. BKA + saline group, analyzed by one-way ANOVA with S-N-K t-test. CV, central vein.

Moreover, immuno-histochemical staining showed consistent changes in the protein expression of the key markers (Figure 4). High-dose BKA treatment significantly elevated the protein levels of CCR1, CCL6, p67, NET, CD11b, MPO, IL-1β, VCAM-1, NT, caspase-3 (active form), and NLRP3. These increases were notably reduced by treatment with NRICM102, NAC, or a combination of both.

BKA disrupts mitochondrial function by inhibiting adenine nucleotide translocation (Anwar et al., 2017). In humans, ANT comprises four isoforms in the mitochondrial carrier family (*SLC25*): AAC1 (*SLC25A4*), AAC2 (*SLC25A5*), AAC3 (*SLC25A6*), and AAC4 (*SLC25A31*). The related proteins UCP5 (BMCP1, encoded by *SLC25A14*) and UCP6 (KMCP1, encoded by *SLC25A30*) are members of this family. Our study found stable expression levels of AAC1, AAC2, and BMCP1 across all groups, whereas KMCP1 was significantly downregulated by BKA (−2.143-fold) and remained unaffected by the treatments.

Liver detoxification of BKA relies on cytochrome P450 (CYP) enzymes (Zhao et al., 2021). Our findings identified significant upregulation of CYP2j8, CYP2t4, CYP4a31, and CYP2c55, and downregulation of CYP2b13a and CYP3a41a following BKA exposure (Table 2). Treatment with NRICM102, NAC, or their combination reversed these changes, indicating potential modulation of CYP activity as a therapeutic mechanism. RNA-Seq also demonstrated that BKA suppressed the expression of critical solute carrier proteins GLUT5 (SLC2a5) and OAT2 (SLC22a7), essential for fructose absorption and organic anion detoxification. Notably, NRICM102, but not NAC, reversed their expression, suggesting that SLC protein downregulation contributes to BKA-induced multiorgan dysfunction (Table 3).

**TABLE 2:** DEG analysis of BKA-induced *CYP* gene alterations in mice treated with NRICM102, NAC, or their combination.

*CYP* enzyme (UP by BKA)	Log_2_ fold change			
	BKA/Control	BKA + 102/BKA	BKA + NAC/BKA	BKA + 102 + NAC/BKA
*CYP2j8*	4.935	−1.496	NA	−0.310
*CYP2t4*	2.835	−1.597	NA	−1.003
*CYP4a31*	2.433	−0.593	−0.023	−2.491
*CYP2c55*	1.975	−1.589	−1.422	−2.916
*CYP* enzyme (DW by BKA)				
*CYP2b13*	−4.759	2.286	1.278	1.498
*CYP3a41a*	−5.462	2.907	0.718	−1.198

^a^ NA: data not available. Italic symbols with underline indicate that the combined effect of NRICM102 and NAC, is more effective in counteracting the effects of BKA, than either NRICM102 or NAC, alone; UP, upregulated by BKA; DW, downregulated by BKA.

**TABLE 3:** DEG analysis showed that BKA-induced solute carrier family (SLC) gene changes were effectively reversed by NRICM102.

Solute carrier family	Log_2_ Fold Change BKA/control	Log_2_ Fold Change BKA + 102/BKA	Log_2_ Fold Change BKA + NAC/BKA	Log_2_ Fold Change BKA + 102 + NAC/BKA
*SLC2a5* (GLUT5)	−2.686	1.965	0.937	0.828
*SLC22a7* (OAT2)	−2.992	1.451	−1.082	−1.700

### 3.4 Effect of NRICM102 on BKA-induced mitochondrial membrane dysfunction in hepatic H4IIE and cardiac H9C2 cells

**FIGURE 5:**
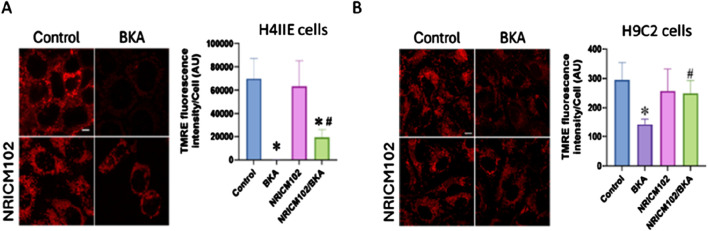
NRICM102 mitigates BKA-induced cytotoxicity and mitochondrial dysfunction in cardiac and hepatic cells. H9C2 cardiac cells and H4IIE hepatic cells were treated with BKA in the presence or absence of NRICM102 and NAC. NRICM102 significantly improved cell viability and preserved mitochondrial membrane potential against BKA-induced damage in both cell types. **(A)** H9C2 cells were pretreated with NAC (4 mM) or NRICM102 (10 μg/mL) for 72 h **(B)** H4IIE cells were pretreated with NAC (2 mM) or NRICM102 at concentrations ranging from 35 to 140 μg/mL for 72 h. Cell viability was evaluated by the MTT assay, while mitochondrial membrane potential was assessed through fluorescence microscopy using TMRE staining (scale bar: 5 μm). Data represent mean ± SEM. Statistical analysis was performed using one-way ANOVA. *p < 0.05 vs. control; ^#^ *p* < 0.05 vs. BKA alone.

Our results demonstrated that NRICM102, combined with NAC, effectively reduced BKA-induced hepatotoxicity and mortality in mice. While NAC is recognized for its antioxidant properties that protect mitochondria-dependent organs (such as the liver and heart) from damage, the mechanism underlying NRICM102s protective effects remains unclear. To clarify this, we assessed mitochondrial membrane potential using TMRE fluorescence intensity in hepatic (H4IIE) and cardiac (H9C2) cell lines exposed to BKA. NRICM102 significantly attenuated the BKA-induced reduction in mitochondrial membrane potential in both H4IIE cells (Figure 5A; F3, 13 = 24.5) and H9C2 cells (Figure 5B; F3, 12 = 6.0). These findings indicate that NRICM102 helps preserve mitochondrial membrane integrity and function, suggesting a possible mechanism for its protective effects against BKA-induced toxicity. While TMRE assays indicate improved mitochondrial membrane potential, we cannot conclusively demonstrate direct mitochondrial targeting by NRICM102. To address this, we propose future validation using mitochondrial-specific modulators or siRNA knockdown of ANT isoforms and KMCP1 to elucidate direct mechanisms.

## 4 Discussion

Our study is the first to demonstrate the protective effects of NRICM102, alone or combined with NAC, against BKA-induced hepatorenal toxicity (modeled as MOF) in a murine model. NRICM102 (1.5–3.0 g/kg, orally, single administration) significantly improved liver and kidney function, demonstrating stronger effects compared to NAC alone. Combined treatment with NRICM102 and NAC provided additional protection, evident by reduced serum markers (GOT, GPT, and BUN) and increased survival rates (from 20% to 80%–100%). These findings suggest that NRICM102 and NAC may mitigate oxidative stress, inflammation, mitochondrial dysfunction, and cell death; however, these interpretations are associative, and further mechanistic validation is required.

BKA toxicity is driven by the release of damage-associated molecular patterns (DAMPs), such as mitochondrial DNA, activating inflammatory pathways (Woolbright and Jaeschke, 2017). This activation notably involves TLR8 and RAGE receptors, triggering downstream inflammatory signaling pathways (NF-κB, MAPK, NLRP3 inflammasome) and subsequent cytokine production (e.g., IL-1β, IL-6, TNF-α) and reactive oxygen species (ROS). Our RNA-seq and immunohistochemistry (IHC) analyses suggest that NRICM102 may attenuate these inflammatory responses through modulation of IL-1β, NLRP3, and cytochrome P450 enzyme activity related to detoxification. Nevertheless, these findings remain correlative, and targeted mechanistic studies are needed to establish causality.

Previous research identified BKA-induced formation of neutrophil extracellular traps (NETs), which contribute significantly to organ dysfunction and inflammatory pathology (Papayannopoulos, 2018; Ravindran et al., 2019; Zhang et al., 2023; Zhou et al., 2021). While our results indicate that NRICM102 treatment is associated with reduced NET-related inflammation and oxidative stress, particularly involving pathways like CD14 and chemokine receptor interactions, additional investigations employing NET-specific inhibitors or genetic models are required to substantiate direct mechanistic links.

BKA is a highly unsaturated tricarboxylic fatty acid that disrupts cellular energy production by inhibiting adenine nucleotide translocase (ANT), a critical mitochondrial protein. Humans possess four ANT isoforms (AAC1, AAC2, AAC3, AAC4) within the mitochondrial carrier protein family (*SLC25*) (Kunji et al., 2020; Ruprecht and Kunji, 2020). Members like UCP5 (BMCP1) and UCP6 (KMCP1) also belong to this protein family (Gorgoglione et al., 2019). Our findings revealed significant suppression of mitochondrial carrier protein KMCP1 (SLC25A30) expression by BKA, partially restored by NRICM102 treatment (Haguenauer et al., 2005). While these RNA-seq data suggest a beneficial role of NRICM102 in maintaining mitochondrial integrity, they remain associative. Future experiments utilizing mitochondrial-specific inhibitors or gene knockdown approaches are needed to confirm direct effects.

Additionally, mitochondrial NAD^+^ transport (MCART1, SLC25A51) (Kory et al., 2020; Luongo et al., 2020) and glutamate transport (GC1, SLC25A22) (Goubert et al., 2017; Casimir et al., 2009), crucial for energy metabolism and detoxification, were upregulated by BKA exposure, possibly as compensatory mechanisms. NRICM102 and NAC treatment correlated with normalization of these transporter expressions, potentially preserving mitochondrial function in cardiac and hepatic tissues. However, direct regulatory mechanisms must be confirmed through further targeted research.

Our RNA-seq data further highlighted that NRICM102 and NAC treatment correlated with restored expression of essential solute carriers (SLC2A5, SLC22A7) (Bumpus and Johnson, 2011; Song et al., 2023), potentially contributing to organ protection. Similarly, modulation of specific CYP enzymes (CYP2j8, CYP2t4, CYP4a31, CYP2c55, CYP2b13, and CYP3a41a) (Barone et al., 2009; Bumpus and Johnson, 2011; Graves et al., 2013; Zhao et al., 2021) by the combination treatment suggests possible normalization of oxidative stress and toxin detoxification processes. However, these relationships remain correlative; direct causal evidence requires additional experimental validation. Overall, NRICM102s anti-inflammatory potential and NAC’s antioxidant actions suggest complementary roles in managing toxin-induced multi-organ failure. This integrative therapeutic approach offers a promising direction; however, future mechanistic validations and preclinical studies are crucial for confirming clinical applicability and safety.

### 4.1 Limitations and future directions

Our study demonstrates that the combination of NRICM102 with NAC effectively reduces BKA-induced toxicity, improving survival rates and attenuating hepatic damage in mouse models. However, several limitations should be acknowledged. Firstly, significant gaps remain in understanding precisely how NRICM102 and NAC counteract systemic toxic effects induced by BKA, particularly regarding dysregulated immune responses and multi-organ failure mechanisms. Although our transcriptomic and histological findings indicate involvement of inflammatory and mitochondrial apoptotic pathways, the detailed molecular mechanisms underlying these protective effects require further investigation. Secondly, comprehensive pharmacokinetic profiling of NRICM102 and identification of its key bioactive components are necessary to facilitate its translation into clinical practice. Finally, while our current focus was on liver protection, additional effects on other mitochondria-rich organs such as the heart, critical in the context of multi-organ failure, remain unexplored. Future studies should clarify these molecular mechanisms, investigate organ-specific protective effects beyond hepatic outcomes, and rigorously evaluate clinical feasibility. Addressing these areas will solidify the mechanistic understanding and aid in developing NRICM102 combined with NAC as a robust and effective TCM-based therapeutic strategy for managing BKA poisoning.

## 5 Conclusion

Our study demonstrates that NRICM102 combined with NAC effectively mitigates severe bongkrekic acid toxicity, significantly improving survival and reducing organ damage in mouse models. Molecular analyses suggest modulation of inflammatory and metabolic pathways. Given the recent fatal BKA outbreak in Taiwan and the urgent need for effective treatments, our findings present a promising therapeutic approach. Future research should focus on mechanistic validation through mitochondrial-targeted studies, as well as preclinical safety and pharmacokinetic evaluations, to expedite clinical translation.

## Data Availability

The data presented in the study are deposited in the NCBI (BioProject) repository, accession number PRJNA1267248.
