# Supplementary material for: Synergistic protective effects of TCM formula NRICM102 and N-acetylcysteine against hepatorenal injury in a mouse model of bongkrekic acid poisoning
Source: Front Pharmacol. 2025 Jun 5;16:1596785. doi: 10.3389/fphar.2025.1596785 (PMC12177335; doi:10.3389/fphar.2025.1596785)
Supplement: Supplementary file 1 [file DataSheet1.docx]

**Supplementary Information**

**Synergistic Role of TCM formula NRICM102 and NAC in Reducing Hepatotoxicity and Renal Damage in Bongkrekic Acid Poisoning in a Mouse Model**

Yuh-Chiang Shen^1,2,^*, Yea-Hwey Wang^2^, Kuo-Tong Liou^1,3,4^, Wen-Chi Wei^1^, Jing-Jy Cheng^1^, Hui-Kang Liu^1^, Nai-Kuei Huang^1^, I-Wen Lo^1^, Cher-Chia Chang^1,5^, Wen-Fei Chiou^1^, Keng-Chang Tsai^1,6^, Chun-Tang Chiou^1^, Chia-Ching Liaw^1,7,8,9,^*, Yi-Chang Su^1,^*

*^1^ National Research Institute of Chinese Medicine, Ministry of Health and Welfare, Taipei City 112026, Taiwan*

*^2^ National Taipei University of Nursing and Health Science, Taipei City 112303, Taiwan*

*^3^ Department of Medicine, Mackay Medical College, New Taipei City 252005, Taiwan*

*^4^ Department of Chinese Medicine, Tri-Service General Hospital, National Defense Medical Center, Taipei City 114202, Taiwan*

*^5^ Institute of Pharmacology, School of Medicine, National Yang Ming Chiao Tung University, Taipei City 112304, Taiwan*

*^6^ Ph.D. Program in Medical Biotechnology, College of Medical Science and Technology, Taipei Medical University, Taipei City 110301, Taiwan*

*^7^ Department of Biochemical Science and Technology, National Chiayi University, Chiayi 600, Taiwan*

*^8^ Department of Pharmacy, School of Pharmaceutical Sciences, National Yang Ming Chiao Tung University, Taipei 112304, Taiwan*

*^9^ Graduate Institute of Natural Products, Kaohsiung Medical University, Kaohsiung 807378, Taiwan*

* Corresponding authors at National Research Institute of Chinese Medicine, MOHW, Taiwan. Address: [No. 155-1, Sec. 2, Linong St., Beitou District, Taipei 112026, Taiwan](https://translate.google.com/website?sl=zh-CN&tl=en&hl=zh-TW&client=webapp&u=https://www.google.com/maps?ll%3D24.156076,120.680367%26z%3D15%26t%3Dm%26hl%3Dzh-TW%26gl%3DTW%26mapclient%3Dembed%26cid%3D11977439491422085888). E-mail: sychang@nricm.edu.tw (Y.-C. Su), Tel +886-2-28201999 ext. 3101; E-mail: ychcs@nricm.edu.tw (Y.-C. Shen), Tel +886-2-28201999 ext.9101. E-mail: liawcc@nricm.edu.tw (C.-C. Liaw), Tel +886-2-28201999 ext.2621.

**Content**

**Figure S1.** HPLC fingerprints of NRICM102 with twenty compounds.

**Table S1.** Liver-to-Body Weight Ratio in the Acute BKA Model (5.0 mg/kg)

**Table S2.** Changes in Body Weight During BKA 2.0 (Subacute Phase)


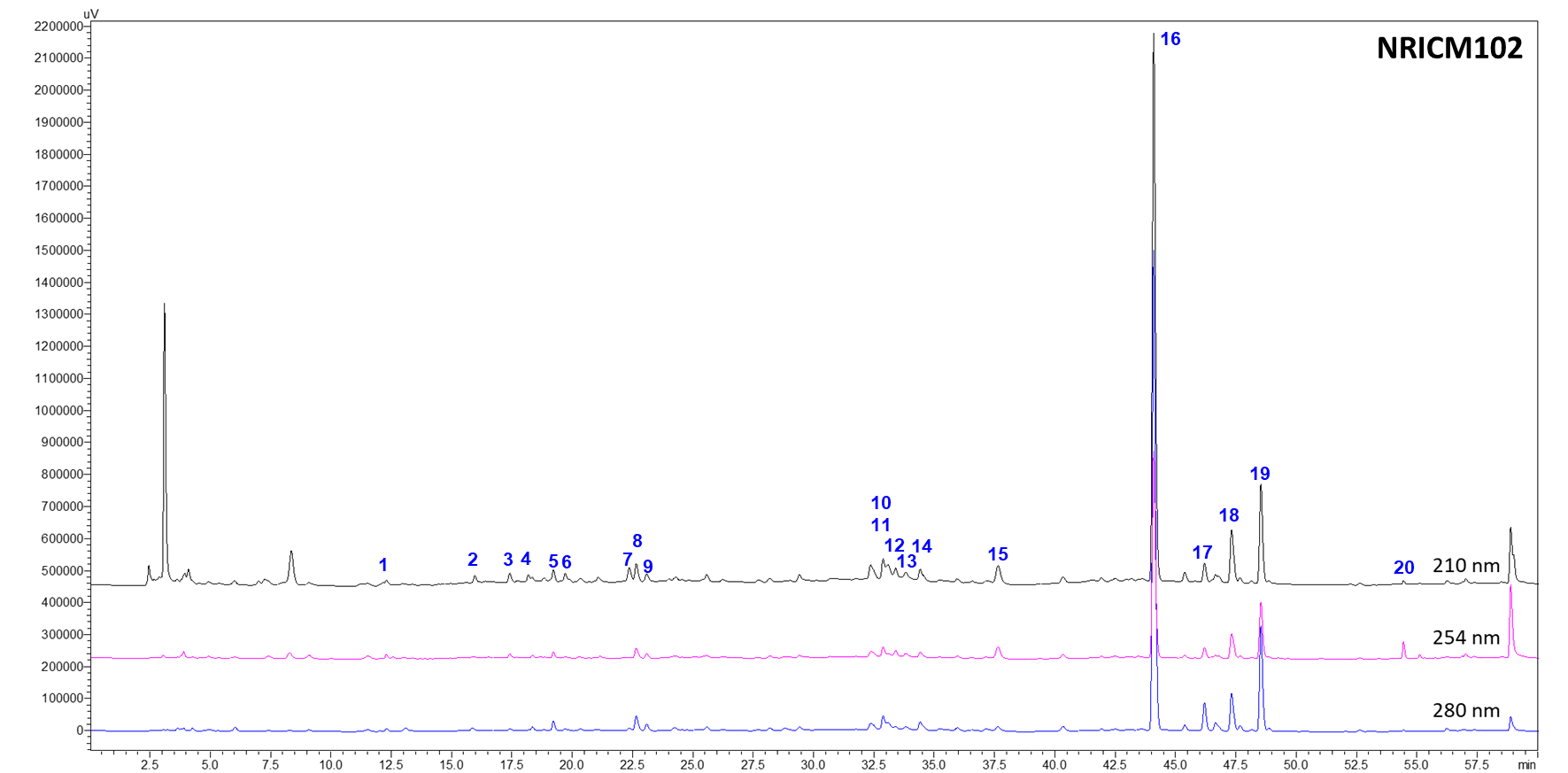


**Figure S1.** HPLC fingerprints of NRICM102 with twenty compounds. **1**: Gallic acid; **2**: *L*-Phenyalanine; **3**: Protocatechuic acid; **4**: Protocatechuic acid 3-glucoside; **5**: 5-Chlorogenic acid; **6**: *L*-Tryptophan; **7**: *L*-Tyrosine; **8**: 3-Chlorogenic acid; **9**: 4-Chlorogenic acid; **10**: Chrysin 6-*C*-arabinoside 8-*C*-glucoside; **11**: Liquirtin; **12**: Quercetin 3-galactoside; **13**: Quercetin 3-glucoside; **14**: Chrysin 6-*C*-glucoside 8-*C*-arabinoside; **15**: Quercetin 3-rhamnoside; **16**: Baicalin; **17**: Norwogonin 7-glucoside; **18**: Oroxyloside; **19**: Wogonoside; **20**: Glycyrrhizic acid.

**Figure S2. A heatmap plot of DEGs**


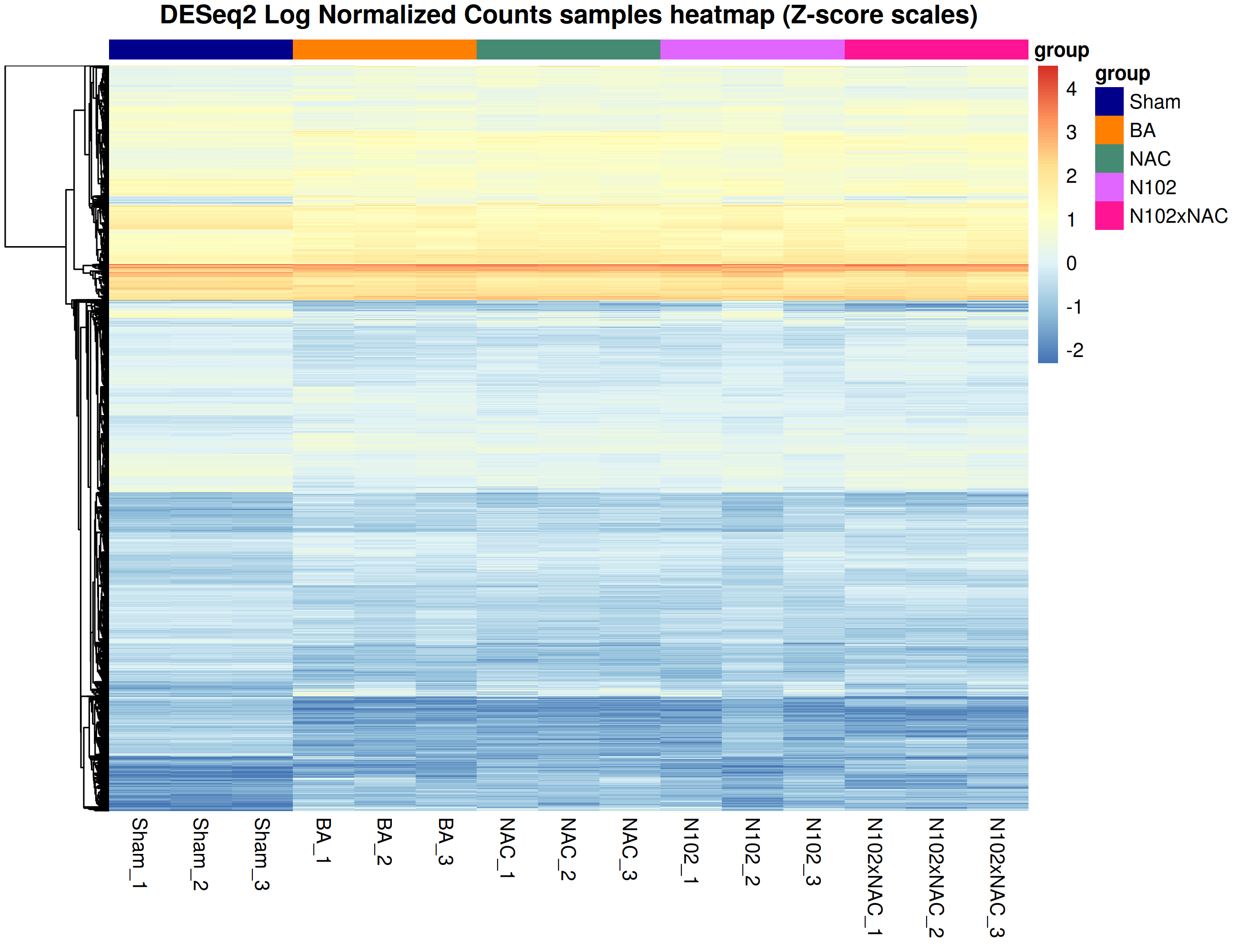


Grouping: Sham (Control), BA (BKA_5.0mg/kg PO), N102 (BKA_ 5mg/kg PO +NRICM102_3.0g/kg), NAC (BKA_5.0 mg/kg PO +NAC_1.5g/kg), N102+NAC (BKA_5.0mg/kg +NAC_1.5g/kg+NRICM102_3.0g/kg).

**Figure S3. PCA analysis of DEGs**


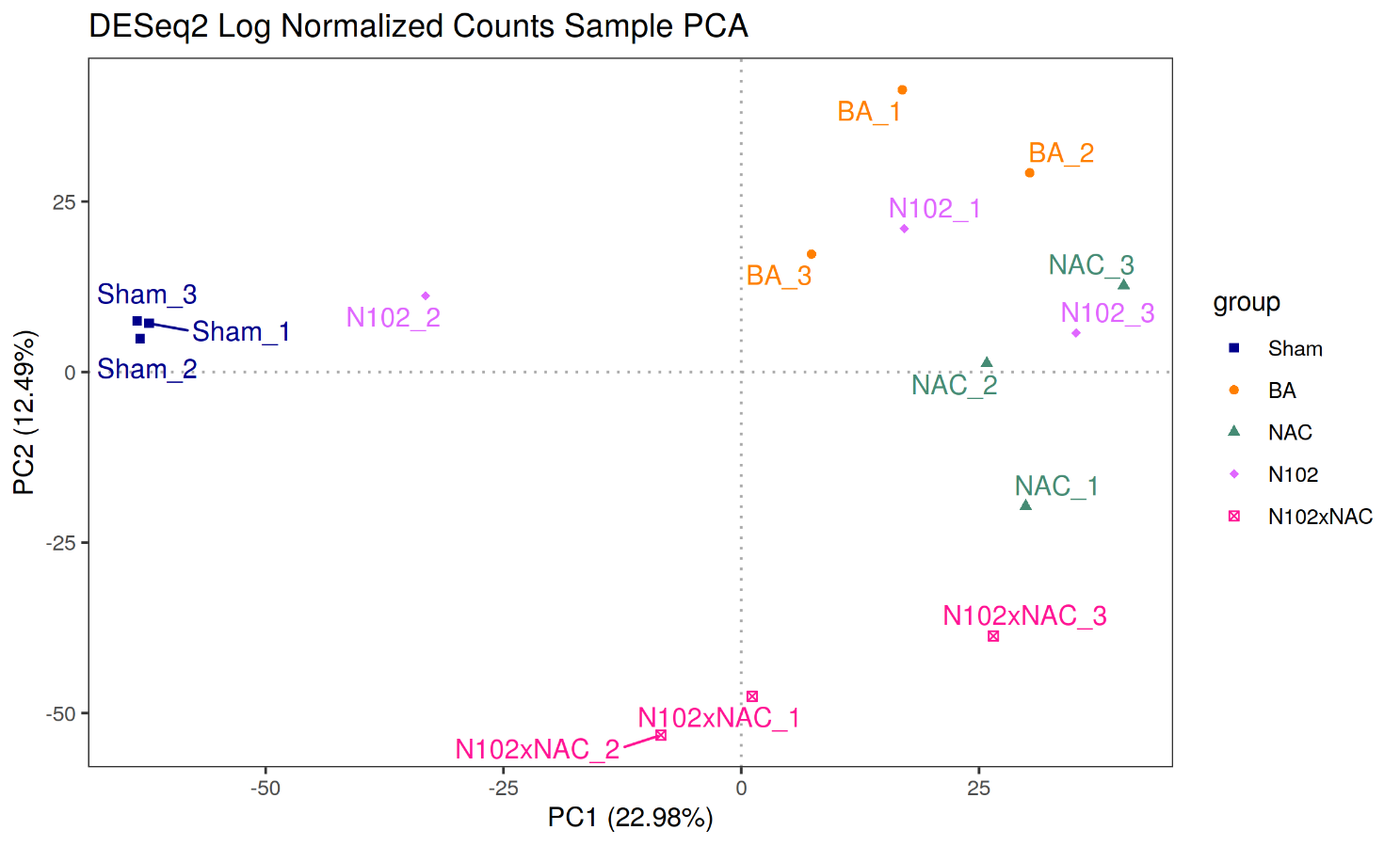


Grouping: Sham (Control), BA (BKA_5.0mg/kg PO), N102 (BKA_ 5mg/kg PO +NRICM102_3.0g/kg), NAC (BKA_5.0 mg/kg PO +NAC_1.5g/kg), N102+NAC (BKA_5.0mg/kg +NAC_1.5g/kg+NRICM102_3.0g/kg).

**Table S1.** Liver-to-Body Weight Ratio in the Acute BKA Model (5.0 mg/kg)

| Group | Liver (g) | B.W. (g) | Liver/B.W. (%) | Mean  S.E.M. | *P*<0.05 |
| --- | --- | --- | --- | --- | --- |
| BA_5.0mg/kg PO | 1.18 | 28.1 | 4.20 | 4.28 | No |
|  | 1.19 | 27.3 | 4.36 | 0.08 |  |
| BA+102_3.0g/kg | 1.09 | 28.1 | 3.88 | 3.96 | No |
|  | 1.14 | 28.2 | 4.04 | 0.08 |  |
| BA+102_1.5g/kg | 1.12 | 27.4 | 4.09 | 3.98 | No |
|  | 1.07 | 27.6 | 3.88 | 0.11 |  |
| BA+NAC_1.5g/kg | 1.15 | 27.9 | 4.12 | 4.04 | No |
|  | 1.11 | 28.1 | 3.95 | 0.09 |  |
| BA+NAC_1.5g/kg+102_3.0g/kg | 1.16 | 28.1 | 4.13 | 4.14 | No |
|  | 1.16 | 27.9 | 4.16 | 0.01 |  |

Liver-to-body weight ratios (%) were measured in five experimental groups, each consisting of two animals (n=2). Due to the small sample size, a non-parametric Kruskal–Wallis H test was used to assess statistical differences among groups. Although trends were observed, the test did not identify any statistically significant differences (**H = 7.31**, p = 0.120). Data are presented as individual values. Given the limited sample size, these results should be interpreted as exploratory, and further validation in larger cohorts is warranted.

**Table S2.** Changes in Body Weight During BKA 2.0 (Subacute Phase)

| Group/day | BKA+saline | BKA+102H | BKA+102L | BKA+NAC | *P*<0.05 |
| --- | --- | --- | --- | --- | --- |
| Day 0 | 27.7± 0.2 | 27.7 ± 0.3 | 27.6 ± 0.2 | 27.9 ± 0.2 | no |
| Day 14 | 29.4 ± 0.3 | 30.3 ± 0.3 | 29.9 ± 0.4 | 29.7 ± 0.2 | no |
| Day 21 | 31.6 ± 0.1 | 32.3 ± 0.2 | 32.1 ± 0.2 | 32.2 ± 0.2 | no |

n=4, each group.
